# Long-Term Fitness Profile and Performance-Related Indicators in Semi-Professional Female Handball Players

**DOI:** 10.3390/sports14100469

**Published:** 2026-10-21

**Authors:** Antanas Skarbalius

**Affiliations:** Department of Coaching Science, Lithuanian Sports University, LT44221 Kaunas, Lithuania; antanas.skarbalius@lsu.lt

**Keywords:** team handball, female athletes, athlete longitudinal monitoring, physiological assessment, playing position, normative values, game-quality performance

## Abstract

Long-term, single-club datasets linking fitness and match performance are rare in women’s handball. This retrospective study profiled fitness and game-quality indicators across 14 seasons (2010–2022, 2026; N = 45; 8–17 players/season). Anthropometric and specific-fitness values (height 173.90 ± 5.53 cm, body mass 68.28 ± 8.08 kg, jump-throw velocity 79.94 ± 6.83 km/h, countermovement jump 43.46 ± 4.84 cm, VO_2_max 48.01 ± 3.61 mL/kg/min) were sub-elite. Shot efficiency (55.74 ± 6.00%) approached elite values (58.90 ± 7.60%). Backs were older, more experienced, and higher-scoring than Wings and Pivots. Universal players matched Backs’ output despite being younger and less experienced. Experience correlated with goals per match (r = 0.51, *p* = 0.001), positive actions (r = 0.44, *p* = 0.005), and action balance (r = 0.47, *p* = 0.003). Throwing velocity and sprint/agility speed showed similar associations. Endurance, strength, and coordination/dribble deficit showed none. Fitness indicators improved across 2010–2022, but a single season after near-complete roster turnover departed markedly from this trend. Trends should not be extrapolated to a changed roster without retesting. A small set of trainable, sport-specific qualities, not general fitness, best predicts match performance. Findings provide normative benchmarks and training priorities for coaches.

## 1. Introduction

Physical fitness and sports performance testing is now routine in handball athlete monitoring [1,2]. Several reviews have synthesized the anthropometric, physiological, and performance characteristics of handball players across sexes and competitive levels [3,4,5]. Yet even these syntheses show that the link between fitness qualities and actual match performance remains inconsistent. A systematic review of female, team-based ball-sport athletes found that evidence linking fitness to sport-specific technical skills is limited and heterogeneous [6]. This is a critical gap. A fitness profile that cannot be linked to competitive output offers limited guidance for training. Its predictive relevance for game performance remains largely unconfirmed in women’s handball.

Assessed fitness attributes span several domains. Sport-specific qualities, particularly ball-throwing velocity, have been linked to anthropometric characteristics in elite women’s handball [7]. Other work has related specific, handball-derived tests to general athletic test batteries [8]. General athletic qualities, including speed, power, agility, strength, and endurance, have been profiled extensively in elite team-handball players [9]. These qualities differ by playing position, competition level [10,11], and sex [12]. Locomotor demands and their match-induced impairment have also been characterized in elite male players [13]. Body composition, height, body mass, BMI, fat-free mass, and fat mass are routinely recorded in athlete screening. Anthropometric characteristics were shown to influence technical activity and playing performance in female elite team handball [14], and a similar relationship was reported in adolescent players [15]. Despite this evidence, direct links between fitness domains and concrete match-performance indicators, goals per match, shooting efficiency, or the balance of positive and negative actions, remain scarce outside elite-level studies, and are essentially absent for semi-professional female handball.

This gap is compounded by the fact that most evidence originates from elite or youth cohorts [16,17]. Semi-professional and amateur levels remain under-represented, despite documented differences in physical performance across competitive levels and positions [18]. Comprehensive testing across specific, athletic, and body-composition domains is time- and resource-intensive. Semi-professional programs typically have more limited staff, equipment, and access time to players than elite programs.

A further, largely unaddressed issue concerns the ecological validity of fitness–performance relationships. Much existing evidence derives from single-season, cross-sectional, or short-term designs, often pooling players across teams and coaching philosophies. Longitudinal designs tracking the same team across a full season remain rare even at the elite level [19]. Multi-season, single-club designs are essentially absent from the handball literature. Such short-duration, mixed-sample designs are vulnerable to confounding by season-level trends and coaching turnover. They rarely allow for fitness–performance relationships to be verified within a stable, consistent environment. A long-term, single-club design offers a rare opportunity to examine these relationships with much higher ecological validity. In such a design, the same target population is repeatedly assessed under one continuous coaching philosophy across multiple seasons.

Taken together, these gaps define three interconnected problems this study addresses. First, from a practical standpoint, it remains unclear which of the many fitness indicators commonly assessed in handball are actually and consistently related to game-quality performance at the semi-professional level. Second, from a methodological standpoint, much existing evidence on fitness–performance relationships is drawn from short-term or cross-sectional designs, or from team-level trends aggregated over time. Both are vulnerable to an ecological correlation problem. Associations observed at the team or season level do not necessarily reflect true individual-level relationships, particularly when both variables share a common temporal trend. Third, from the standpoint of data value, long-term, single-club datasets tracking the same target population under a stable coaching philosophy across more than a decade are exceptionally rare in handball. They are essentially absent for semi-professional women’s teams. This limits the field’s ability to study fitness–performance relationships under ecologically consistent conditions.

Building on these considerations, this study aimed to characterize the long-term fitness profile of a semi-professional female handball team. The team was tracked across 14 competitive seasons. The study also examined this profile’s relationship with game-quality performance. The study pursued five objectives. First, establish normative values, including 95% confidence intervals and percentiles, for the fitness and game-quality indicators in this population. Second, compare fitness and game-quality indicators among four outfield positions (backs, wings, pivots, universal players); goalkeepers are described separately given their distinct demands. Third, determine which fitness-related indicators, specific, athletic, and body-composition, are most strongly and consistently associated with four game-quality indicators (goals per match, shooting efficiency, positive actions, negative actions). Fourth, examine relationships between age, playing experience (seasons), and the same game-quality indicators. Fifth, examine changes in fitness and game-quality indicators across the 14-year period. It was hypothesized that playing experience and handball-specific abilities would be the strongest determinants of match performance in this population.

## 2. Materials and Methods

### 2.1. Study Design and Participants

**Figure 1:**
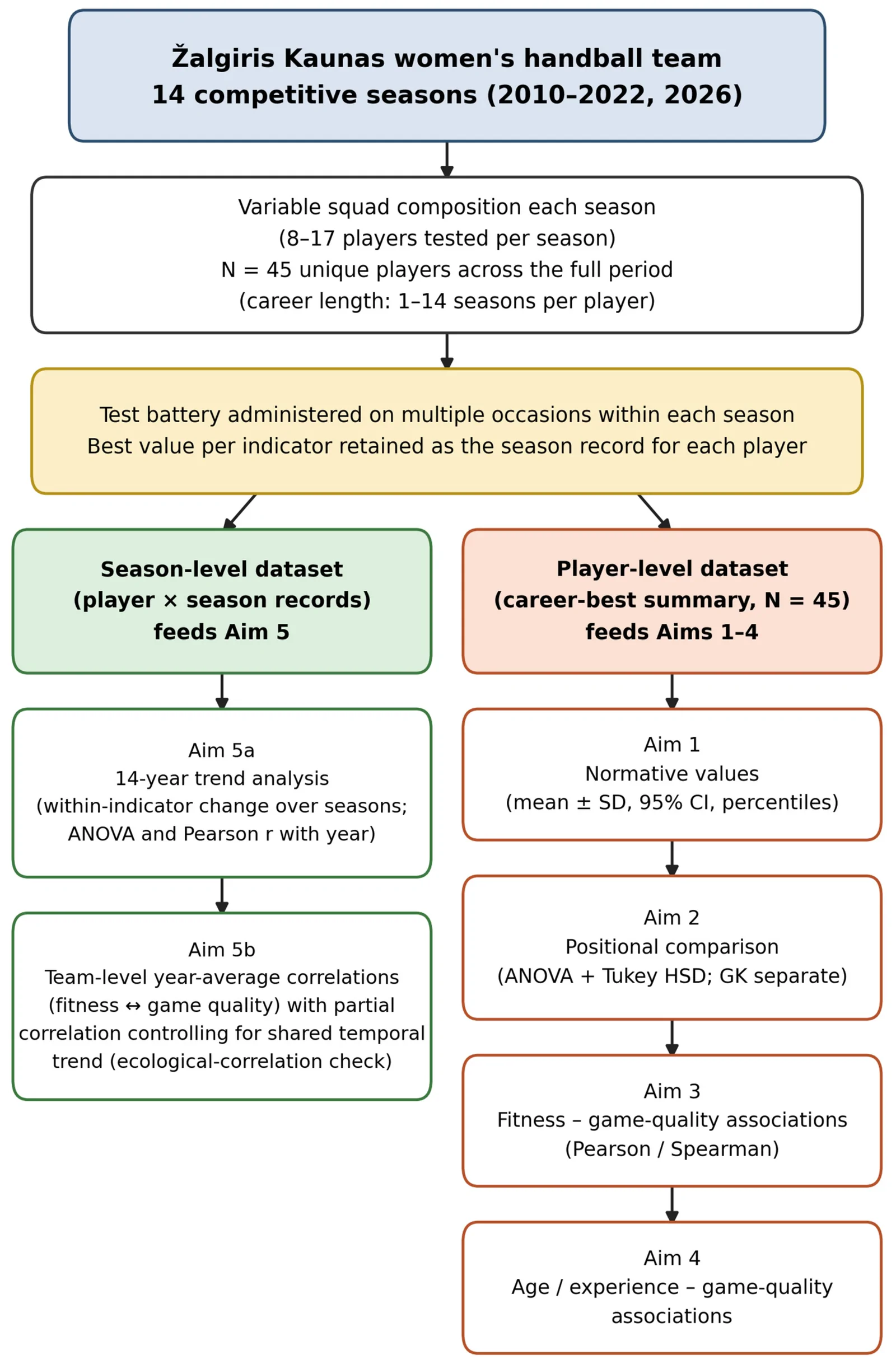
Study design and analytical workflow, showing squad turnover across the 14 seasons, the within-season best-value selection procedure, and the two resulting datasets used across the five study aims.

This study used a retrospective, longitudinal, single-club design. It characterized the long-term fitness profile of a semi-professional female handball team and examined its relationship with game-quality performance. Data were collected across 14 competitive seasons (2010–2022 and 2026) from the same club, Žalgiris Kaunas, under a continuous coaching philosophy. Squad composition varied from season to season, with 8–17 players tested per season. Across the full 14-season period, 45 unique players were included, with career lengths ranging from 1 to 14 seasons. Two complementary data structures were used. A season-level dataset (one record per player per season) was used to examine change over the 14-year period. A player-level dataset (one summary record per player, N = 45) was used to examine fitness–game-quality relationships, age/experience effects, normative values, and positional differences (Section 2.3, Figure 1). Goalkeepers (n = 7) were analyzed separately throughout. The remaining 38 outfield players were categorized into four positions: backs (n = 14), wings (n = 14), pivots (n = 3), and universal players (n = 7).

The study was approved by the Regional Biomedical Research Ethics Committee (No. BE-2-55) and conducted in accordance with the Declaration of Helsinki.

### 2.2. Test Battery and Data Collection

Players underwent a standardized battery of anthropometric, physical, and sport-specific tests, administered on multiple occasions each season (Figure 1); testing was integrated into the players’ regular training. Body-composition and strength indicators were consistently assessed at the start of each microcycle (Mondays). For each indicator, the best value achieved across a season’s testing occasions was retained as that season’s record, except for body composition, which was recorded at the end of the regular-season stage, immediately before the play-offs (Section 2.2.10). Testing indicators were grouped into ten clusters: (I) player experience (age, seasons); (II) game-quality indicators (goals per match, shooting efficiency, positive actions, negative actions); (III) specific fitness (3-step running throw velocity, jump-throw velocity, 7 m throw velocity); (IV) power (CMJ); (V) speed (5 m, 20 m, and flying 15 m sprint, running and dribbling); (VI) agility (running and dribbling); (VII) coordination (relative difference between dribbling and running times); (VIII) strength (flexed-arm hang, plank hold, 30 s sit-ups, 60 s curl-ups); (IX) endurance (Yo-Yo IE1, 3000 m run, VO_2_max); and (X) body composition (height, body mass, BMI, fat mass, fat-free mass, derived indices). Different tests were administered at different points in the season, depending on the competition calendar.

#### 2.2.1. Player Experience

Player age and playing experience (number of competitive seasons) were recorded once per season, at the end of the regular-season stage of the Lithuanian national championship (April); the ongoing season was included in the player’s cumulative experience total.

#### 2.2.2. Game-Quality Indicators

Handball match performance is shaped by a wide range of playing actions [14,20,21,22,23]. Skarbalius [2] established reference values for match-performance indicators in a semi-professional team; based on this framework, outfield players’ on-court actions were classified into four clusters: (1) goals scored; (2) shooting efficiency; (3) positive actions (forcing a 7 m penalty, steals, assists, breakthroughs, blocked shots, and defensive actions that stopped an opponent’s attack); and (4) negative actions (turnovers, conceding a 7 m penalty, 2 min suspension, and attacking actions stopped by the opposing defense). Turnovers were any loss of ball possession without a shot attempt, including technical errors, offensive fouls, or passive-play/time-limit violations. Each positive action was scored +1 and each negative action −1; overall player quality was evaluated from the sum and from the difference between them. Goalkeepers’ game quality was evaluated separately, using save percentage. Following the procedure described by Skarbalius [2], each outfield player’s match performance was recorded by an assistant coach. Recording was verified against the official match report and an independent shot-registration protocol completed by the substitute goalkeeper, with video analysis used when needed. Final season-level values were calculated as the player’s per-match average.

#### 2.2.3. Specific Fitness: Throw Velocity

Ball-throwing velocity was assessed using a calibrated Doppler radar gun (Stalker Pro, Applied Concepts Inc., Richardson, TX, USA; 100 Hz) (Stalker Pro, Applied Concepts Inc., USA; 100 Hz), positioned behind the goal and aligned with the ball’s trajectory. After a standardized warm-up, players performed three maximal-effort throws: a 9 m three-step running throw, a 9 m three-step jump throw, and a 7 m standing (penalty-style) throw, with 1–2 min passive recovery between trials. Velocity was assessed 3–4 times per season; the highest recorded value (km·h^−1^) for each throw type was retained, as this procedure provides reliable and sensitive assessment of throwing performance in female handball players [24,25].

#### 2.2.4. Power: Countermovement Jump (CMJ)

Lower-limb explosive power was assessed using the countermovement jump (CMJ) test, following the protocol previously described for this cohort [2]. Players performed a maximal vertical jump with self-selected knee-flexion depth and free arm swing, using the Ergojump–Bosco system (Psion XP, MA.GI.CA., Rome, Italy) [26]; this protocol allows for individualized knee angles and natural arm actions, more closely replicating match-play coordination demands. Players had performed this jump for several years and demonstrated highly consistent technique. CMJ was measured at the start of every training session and before matches; the highest value recorded per season was retained.

#### 2.2.5. Running Speed

Running speed was assessed over three distances relevant to handball’s short, repeated sprint demands [3,4,5,6,8,9,10,11,12,13,18,23,27,28]: a 5 m sprint, assessing the acceleration phase [8,29]; a 15 m flying sprint, assessing maximal running speed once acceleration was achieved [27]; and a combined 20 m sprint, reflecting handball match-play sprint distances of approximately 20–25 m [28].

Sprint performance was assessed using a Microgate Witty photoelectric timing system (Microgate, Bolzano, Italy), with wireless photocells positioned along a 20 m indoor sprint lane and calibrated before each session. Following a standardized warm-up, players performed maximal 20 m sprints from a standing start, with the starting line 0.5 m behind the first timing gate. Athletes initiated the sprint voluntarily and continued running past the finish line to avoid deceleration before the final gate. Three split times were recorded: 0–5 m, 5–20 m (15 m flying section), and 0–20 m. Running speed (m·s^−1^) was calculated automatically for each section. Each player completed three maximal trials, separated by 3–5 min recovery; the fastest performance per variable was retained.

#### 2.2.6. Boomerang Agility Test

**Figure 2:**
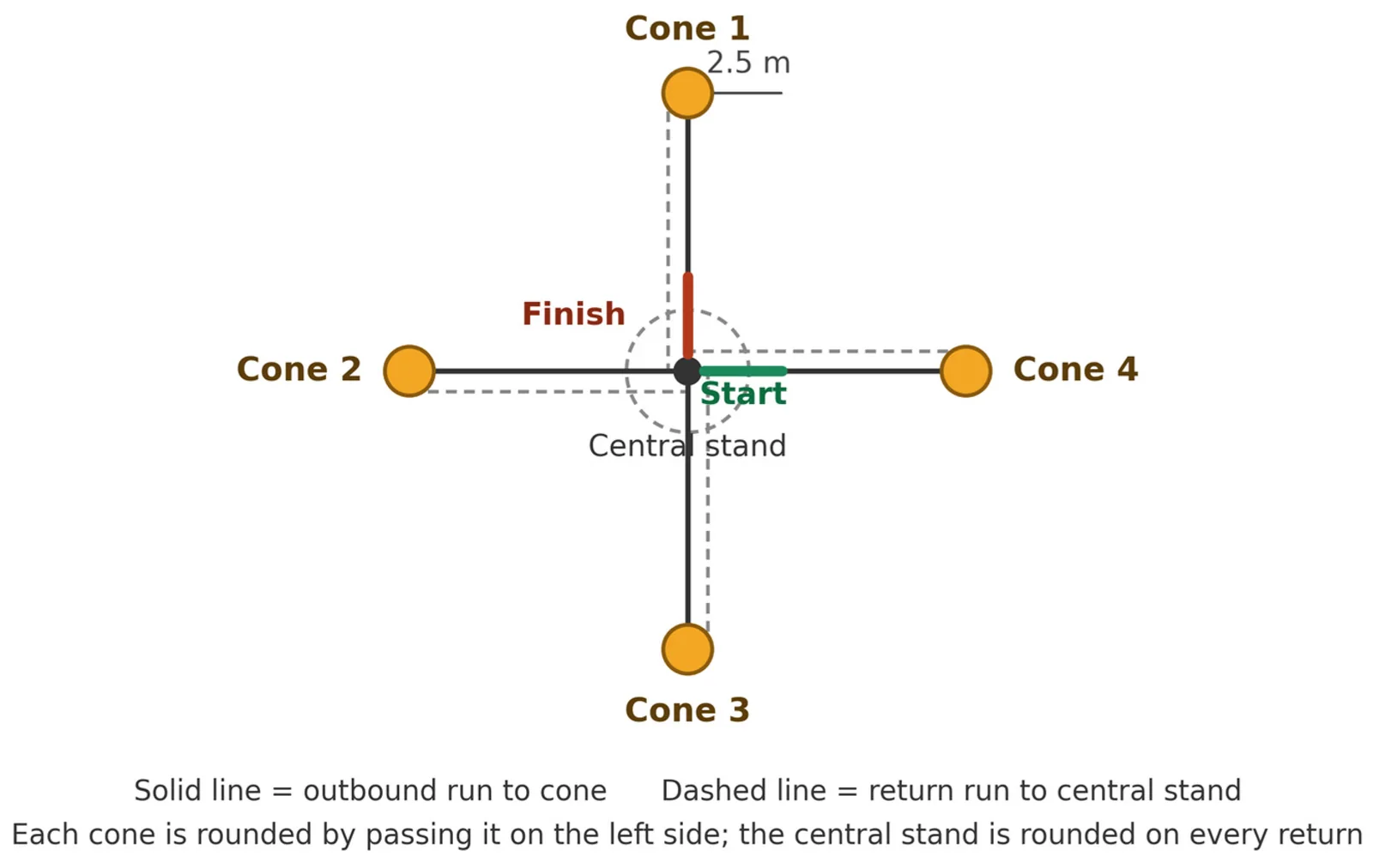
Schematic layout of the boomerang agility test, showing the central stand, the four peripheral stands (Cones 1–4) positioned 2.5 m away in each cardinal direction, and the start/finish lines.

Agility was assessed using the boomerang agility test, which is practically applied by handball coaches and closely resembles on-court movement demands [30,31]. Four peripheral stands were placed 2.5 m from a central stand in each cardinal direction, reflecting the typical range of forward, backward, and lateral movement during match play (Figure 2); a 1 m horizontal line marked the start position and a 1 m vertical line marked the finish position, both adjacent to the central stand.

The test required continuous direction changes. After rounding a numbered stand, the athlete returned to and rounded the central stand before proceeding to the next stand, always passing stands on the left side. On the command “go,” the athlete ran to stand 1, rounded it, returned to and rounded the central stand, then repeated the pattern for stands 2, 3, and 4. After rounding stand 4, the athlete ran to and crossed the central stand’s finish line. Movement was performed without turning sideways; backward movement was permitted only by running backward, and stands were rounded only by moving sideways around them.

Time was recorded by two coaches using handheld stopwatches accurate to 0.01 s; when recorded times did not match, their mean was used. Concurrent validity, assessed against the Illinois agility test in all 45 players each season, was consistently strong (Pearson r = 0.81–0.84 across seasons, all *p* < 0.001). Test–retest reliability was likewise consistently good (ICC = 0.79–0.83, all *p* < 0.001).

#### 2.2.7. Coordination/Dribble Deficit

Coordination is an important component of handball performance, underpinning throwing, jumping, and other handball-specific movements [4,23]. Coordination training has also been shown to improve psychomotor abilities relevant to handball performance in adolescent players [32]. In the present study, sport-specific coordination was assessed using the Dribble Deficit (DD) concept, which quantifies the additional time required to perform a locomotor or agility task while dribbling compared with the same task performed without the ball [33,34]. Because dribbling performance is partly determined by an athlete’s underlying running and agility capacity, expressing the additional time required when ball control is introduced provides an estimate of the performance cost associated with integrating locomotion and ball handling [33].

The Dribble Deficit (DD) was calculated for each test as the percentage difference between the dribbling and running conditions:

DD (%) = [(Tdribbling time − Trunning time)/Trunning time] × 100

where (T_{dribbling}) represents the time required to complete the test while dribbling and (T_{running}) represents the time required to complete the identical test without the ball. This calculation was applied to the 5 m sprint, 20 m sprint, flying 15 m sprint, and Boomerang agility test, with each test performed under both running and dribbling conditions. A composite Dribble Deficit score was calculated as the arithmetic mean of the four percentage differences. Lower DD values indicated a smaller performance decrement when ball control was introduced and therefore greater efficiency in integrating locomotor performance with ball-handling demands.

#### 2.2.8. Strength

Muscular strength and localized muscular endurance were assessed using four field tests, flexed-arm hang, plank hold, 30 s sit-ups, and 60 s curl-ups, following the protocol previously described for this cohort [2].

#### 2.2.9. Endurance

Aerobic endurance was assessed using the Yo-Yo Intermittent Endurance Test Level 1 (Yo-Yo IE1) and a 3000 m running time trial. Maximal oxygen uptake (VO_2_max) was determined directly, using an incremental maximal running test to exhaustion performed in a laboratory setting, following the same protocol previously described for this cohort [2]. This laboratory test was conducted only once per season, at the end of the regular-season stage (April); this single annual value was used for VO_2_max in the analysis.

#### 2.2.10. Anthropometric and Body Composition

Body composition was assessed using the protocol previously described for this cohort [2], via multi-frequency bioelectrical impedance analysis (TANITA, Tokyo, Japan), a method widely applied in sports science to estimate body fat and fat-free mass in team-sport athletes [34]. Measurements were performed under standardized conditions to minimize hydration-related variability: consistently on Mondays at 18:00, with lunch standardized to 14:00–15:00. Body height was measured with a stadiometer to the nearest 0.1 cm. Only values obtained after the regular-season stage (April), immediately before the play-offs, were used for analysis.

### 2.3. Statistical Analysis

Statistical analyses were performed using Python 3.12 (Python Software Foundation, Wilmington, DE, USA), with data management in pandas (2.2.3) and statistics in SciPy (1.15.0) and statsmodels (0.14.4). Descriptive statistics (mean ± SD, 95% CI, percentiles) were calculated for all fitness and game-quality indicators. Relationships between fitness indicators and the five game-quality indicators were assessed using Pearson and Spearman correlations at the player level, using each player’s career best-value summary for fitness indicators and career-average match statistics for game-quality indicators; the same approach was used for age/experience relationships. Positional differences among the four outfield positions were assessed using one-way ANOVA with Tukey HSD post-hoc comparisons; goalkeepers were described separately, using descriptive statistics only. Change over the 14-year period was evaluated using season-level Pearson correlations with year and one-way ANOVA across seasons for each indicator; team-level (year-average) correlations between fitness and game-quality indicators were additionally computed, with partial correlations controlling for the shared temporal trend used to guard against ecological correlation. A multiple regression examined the relationship between body mass and negative actions, controlling for position and experience. Statistical significance was set at *p* < 0.05.

## 3. Results

### 3.1. Sample Characteristics

The studied cohort comprised 45 semi-professional female handball players (Table 1). Outfield players (n = 38) showed a positive-to-negative action balance close to zero, with comparable frequency of both action types. Shot efficiency is reported for outfield players only; the goalkeeper equivalent (save efficiency) is a conceptually different metric (Table 2). Full descriptive statistics are in Table 1.

### 3.2. Positional Differences

One-way ANOVA (Table 3) revealed several significant positional differences (Back, Wing, Pivot, Universal; goalkeepers analyzed separately, Table 2). Backs were the oldest, most experienced, and highest-scoring group. Universal players fell between Backs and the others on age and experience. Universal players and Backs combined the highest positive- and negative-action totals; Wings and Pivots recorded fewer of both. Positions also differed in throwing velocity, jump height, and sprint speed, with Backs generally highest and Universal players intermediate. Wings and Pivots further differed in body fat and two strength indicators, though not significantly pairwise. Coordination and endurance showed no positional differences (Table 3).

Goalkeepers (n = 4–7; Table 2) were described separately, as their game-action profile is not comparable to outfield players. Mean age was 26.21 ± 8.03 years, experience 7.93 ± 7.25 years, similar to the outfield sample. Mean save efficiency was 36.89 ± 5.56%; throwing velocity ranged 78.84–82.75 km/h, and CMJ averaged 42.22 ± 3.81 cm, comparable to outfield players.

### 3.3. Relationships Between Fitness Indicators and Game Performance

Pearson correlation analysis (Table 4; full detail in Supplementary Table S1) identified 33 statistically significant associations (*p* < 0.05) out of 170 fitness–game-quality pairs tested.

The strongest associations involved competitive experience, throwing velocity, and sprint speed. Experience correlated with goals per match (r = 0.51, *p* = 0.001), positive actions (r = 0.44, *p* = 0.005), and action balance (r = 0.46, *p* = 0.003). Throwing velocity was consistently related to performance: jump throw with positive actions (r = 0.50, *p* = 0.001) and goals (r = 0.44, *p* = 0.005); 3-step throw with positive actions (r = 0.48, *p* = 0.002); 7 m throw with positive actions (r = 0.44, *p* = 0.006).

Sprint and agility speed were also linked to performance. With the ball, 20 m and flying 15 m speed correlated with goals per match (both r = 0.46, *p* = 0.004); 20 m speed also correlated with action balance (r = 0.41, *p* = 0.011). Agility times, with and without the ball, were both associated with goals (r = −0.41, *p* = 0.013); faster times meant more goals. CMJ was related only to action balance (r = 0.39, *p* = 0.017).

Body mass was associated with negative actions (r = 0.41, *p* = 0.010), confirmed by regression adjusting for position and experience (β = 0.076, *p* = 0.035); heavier players committed more technical/tactical errors independent of position and experience. Body mass showed no association with shot efficiency once goalkeepers were correctly excluded (r = −0.17, *p* = 0.314); an apparent association in preliminary pooled analysis was an artifact of mixing the two indicators.

Because goalkeepers’ save efficiency is conceptually distinct from outfield shot efficiency, their fitness–performance relationship was examined separately (N = 7). Save efficiency correlated negatively with 5 m sprint speed with ball (r = −0.95, *p* = 0.001), 20 m sprint speed (r = −0.86, *p* = 0.014), flying 15 m speed (r = −0.81, *p* = 0.026), and 30 s sit-ups (r = −0.87, *p* = 0.026, n = 6): faster, stronger goalkeepers tended to show lower save efficiency. Given the very small sample, these associations are descriptive, not robust findings.

Because none of the four coordination indicators reached significance (Supplementary Table S1), we tested a composite score (mean of Coordination 5 m, 20 m, flying 15 m, and agility, %). The composite showed a nominally significant correlation with goals per match (r = −0.35, *p* = 0.032, N = 38), but no other association. This did not hold up under scrutiny: the Spearman correlation was not significant (*p* = 0.105), and removing one high-scoring outlier reduced the Pearson correlation to non-significance (r = −0.26, *p* = 0.114). We do not interpret the composite score as meaningfully related to game performance.

Although coordination was unrelated to game performance, two of the four coordination indicators were related to competitive experience (N = 44–45). Coordination 20 m and flying 15 m decreased with age (r = −0.38, *p* = 0.010; r = −0.35, *p* = 0.019) and playing experience (r = −0.37, *p* = 0.013; r = −0.32, *p* = 0.033): older, more experienced players showed a smaller running–dribbling gap, though this was unrelated to in-game performance. Each coordination indicator’s strongest correlate was its own with-ball speed measure, reflecting definitional overlap.

No coordination, endurance (Yo-Yo IE1, 3-km run, VO_2_max), or strength indicator (flexed-arm hang, plank, sit-ups, curl-ups) reached significance in relation to any game-quality indicator (all *p* > 0.05). In this sample, general fitness qualities were less discriminant of game performance than experience, throwing velocity, and linear/agility speed.

### 3.4. Changes Across the Continuous 2010–2022 Period, and the 2026 Season Compared Separately

The team was observed continuously for 13 seasons (2010–2022); a 3-year gap followed, then a single further season (2026). A trend fitted across this gap would conflate real change with an unmodeled discontinuity, and the 2026 roster shared only 3 of its 9 players with 2022. Trend statistics were therefore computed using only 2010–2022 (Table 5); 2026 is instead compared descriptively against the trend’s prediction.

Fourteen of the thirty-one indicators tested showed a significant linear trend across 2010–2022 (Table 5). The squad became significantly older and more experienced, paralleled by an increase in positive actions; negative actions did not reach significance over this window. Throwing velocity, CMJ, with-ball speed and agility measures, and several endurance/strength indicators (Yo-Yo IE1, plank, curl-ups) also improved significantly. Coordination 20 m and flying 15 m, non-significant in the previous pooled trend, both improved once 2026 was excluded. No significant trend was found for goals per match, shot efficiency, height, body mass, body fat, most raw speed indicators, VO_2_max, or most strength indicators.

Comparing the isolated 2026 season against the 2010–2022 trend prediction (Table 5) reveals a clear discontinuity. Endurance and strength indicators (Yo-Yo IE1, flexed-arm hang, plank, curl-ups) were markedly worse than predicted; all four coordination indicators were markedly better, most strikingly coordination 5 m and flying 15 m. Positive actions and body composition were somewhat higher than predicted. Since the 2026 roster consisted mostly of new players relative to 2022, this pattern most plausibly reflects a genuinely different playing group, better coordinated but less aerobically conditioned, rather than a continuation of the trend; discussed further in Section 4.

To test whether years with better average fitness also showed better average game performance, yearly team-mean fitness and game-quality indicators were correlated across the 13 continuously observed seasons (2010–2022; Table 6). Only the 22 pairs of 112 tested that were significant at the raw level (*p* < 0.05) are shown; the full set is in Supplementary Table S2.

Because age, experience, activity volume, and body composition all trended upward together over this period, these raw year-level correlations could reflect the shared temporal trend rather than an independent link. Each correlation was recomputed on the residuals after removing the linear trend against year (Table 6). Of the twenty-two raw significant associations, seven remained significant after adjustment, mostly involving dribbling/sprint speed against negative and positive actions. This is a markedly larger, more coherent set of trend-independent associations than in the previous pooled analysis, where only one association survived adjustment, consistent with the 2026 gap year having distorted the ecological correlation structure.

## 4. Discussion

### 4.1. Fitness and Game-Quality Profile

Across the full battery of indicators (Table 1), this squad presents a physical profile broadly typical for the sport [3,5,10,15,17,23], sitting, on most fitness qualities, closer to sub-elite than elite values [3,5,7,12,14,18,19,20,21,24,35,36,37,38]. Anthropometrically, the squad falls within the range reported for senior female handball players generally [3,14,19,38,39], and body composition is similarly unremarkable, consistent with reports that body mass and fat percentage differ little between elite and amateur players [34], even when other fitness qualities differ substantially [23,34,40]. Throwing velocity, CMJ, and aerobic capacity are respectable but below values typically reported for elite players; the literature reports differences of 10–25% for power, speed, and throwing, even when body composition does not differ [5,7,33,34]. Coordination, agility, and strength indicators are reported here primarily as normative reference values (Objective 1).

The game-quality profile presents a different, more interesting picture. Evaluated only against its own competitive level, mean shot efficiency (55.74 ± 6.00%, outfield players) is comparable to elite women’s handball teams. This requires caution: elite players at the Olympic Games have recorded shot efficiency of 58.90 ± 7.60% [39], already exceeding our sample’s mean and reflecting performance against far stronger opposition. This squad’s competitive-activity indicators would likely not hold against elite opposition; the gap would plausibly reflect its overall preparation, in both physical fitness and technical-tactical readiness, rather than either alone.

In summary, this squad’s physical fitness remains sub-elite in absolute terms, while its competitive-activity indicators are level-relative rather than absolute markers of quality, a distinction essential for interpreting the associations that follow.

### 4.2. Role-Specific Demands and the Value of Universal Players

Backs were older, more experienced, and more prolific scorers than Wings and Pivots, possibly reflecting the cognitive–perceptual demands of the back-court role, which requires rapid decision-making under defensive pressure, skills that improve with age and experience [41,42], consistent with handball as a complex, dynamically adaptive game [43].

A notable finding was that Universal players matched Backs in competitive-activity output despite being younger and less experienced, highlighting their importance given semi-professional squads’ limited size and frequent specialist shortages (left back, right back, playmaker, pivot), which require versatile universal players [23]. This solves an ecological constraint of semi-professional handball, though universal players may not always fully match specialists’ position-specific characteristics, a balance deserving further research.

Positional differences in throwing velocity (Backs > Wings, 7 m throw) and vertical jump (Backs > Pivots) align with back-court players relying more on shooting power at distance, while Pivots operate in closer, contact-based play [5,10,12,14,16,18,23]. Aerobic endurance showed no positional differences, fitting handball’s intermittent, high-intensity demands, where roles differ more in movement type than overall aerobic load [5,14,23].

Several indicators (action balance, jump-throw velocity, plank, sit-ups) showed a significant overall ANOVA but no significant pairwise comparison, typical of small, uneven subgroups (Pivot n = 3): the test detects that not all positions are equal but lacks power to identify which pair differs. This should be read as inconclusive, not as evidence of no difference.

Goalkeepers (N = 7) showed save efficiency (36.89 ± 5.56%) and an anthropometric profile comparable to outfield players, aside from greater body mass and height, consistent with the demand for reach and blocking surface [23,44]. Their age and experience were similar to the outfield squad, with a wide spread reflecting the small subgroup, limiting the fitness–performance analysis for this position (Section 4.3).

Taken together, positional fitness demands vary meaningfully by role, particularly shooting-related power and technical involvement, while aerobic capacity remains broadly uniform, underscoring the practical value of universal players in bridging positional gaps under semi-professional squad-size constraints.

### 4.3. Which Fitness Qualities Relate to Game Performance?

The pattern of significant correlations [6] is, in our view, the most practically important finding of this study. Competitive experience, throwing velocity, and sprint/agility speed were consistently associated with game-quality indicators; coordination, endurance, and strength showed none. CMJ related only to overall action balance, suggesting leg power supports a player’s net productive contribution rather than any single action [6,7,31,44].

This does not mean endurance or strength are unimportant for handball [23]; they may function as a necessary baseline rather than a continuously discriminating factor once a working fitness level is reached [5,6]. Throwing velocity, sprint speed, and agility scale more directly with our indicators, consistent with the biomechanical link between shot velocity and scoring, and between sprint/agility speed and creating or closing space in transition [5,6].

A preliminary analysis suggested an association between body mass and shot efficiency, an artifact of pooling goalkeepers’ save percentage with outfield shot efficiency; excluding goalkeepers removed it. We flag this as a caution for handball datasets computing one “efficiency” column across positions. One body-mass relationship did not depend on this artifact: an association with negative actions, confirmed by regression adjusting for position and experience, indicating heavier players committed more errors independent of position and experience.

Because goalkeepers’ save efficiency is not comparable to outfield shot efficiency, their fitness–performance relationship was examined separately (N = 7). Save efficiency correlated negatively with several speed and strength indicators, tentatively suggesting faster, stronger goalkeepers recorded lower efficiency. Given the very small sample, we do not consider this robust; it may reflect an age/experience confound, since goalkeepers here were older and more experienced than outfield players (Section 4.1).

The absence of a coordination–performance relationship deserves comment. Coordination testing is routine in handball monitoring, assumed to capture something beyond speed and agility tests; our results question that assumption for the metric used here, likely because each individual test carries considerable noise. A composite of the four indicators produced one nominally significant correlation with goals per match that did not survive a Spearman retest or removing a single outlier. We consider this null finding reasonably robust, though it may reflect that this metric misses the features of coordination most relevant to game quality.

Coordination was not unrelated to everything: two of the four indicators (coordination 20 m and flying 15 m) decreased with age and experience, meaning older, more experienced players showed a smaller running–dribbling gap, consistent with coordination developing with practice even though it did not translate into better game outcomes here. These same two indicators also improved significantly across 2010–2022 at squad level (Section 4.4).

Taken together, these findings indicate that a small set of trainable, sport-specific qualities, competitive experience, throwing velocity, and linear/agility speed, account for most of the fitness–game-quality relationships in this squad, while general endurance, strength, and coordination as measured here do not, and should not be assumed to transfer to match output without direct evidence.

### 4.4. Age and Experience in Relation to Game-Quality Performance

Age and competitive experience were both positively associated with several game-quality indicators, independent of the fitness associations above. Experience showed the stronger pattern; age a similar but weaker one. Neither was associated with shot efficiency or negative actions, consistent with the coordination findings in Section 4.3, and with evidence that reaction, perception, and anticipation skills improve with age and experience in handball players [41], and that decision-making depends on accumulated game exposure [42]. Age and experience appear to track accumulated game-reading skill, increasing offensive involvement without a corresponding change in per-action precision or error rate.

Overall, these findings support the study’s hypothesis. Competitive experience and handball-specific abilities, throwing velocity and sprint/agility speed, were the strongest and most consistent determinants of game-quality performance; general endurance, strength, and coordination were not. The exception was shot efficiency, unrelated to age, experience, or any fitness indicator. This may depend on factors not measured here, such as shot selection or defensive pressure, an assumption future research should test.

### 4.5. Trends Across the Continuous 2010–2022 Period, and the Discontinuous 2026 Season

The squad was observed continuously for 13 seasons (2010–2022); a 3-year gap followed, then a single further season (2026) with a roster sharing only three of its nine players with 2022. Because a linear trend across such a gap would conflate genuine development with an unmodeled discontinuity, we separated two questions: how the squad changed across 2010–2022, and whether 2026 continues that trajectory or departs from it.

Across 2010–2022, the increase in squad age and experience is consistent with a maturing, settled roster. This makes the parallel increase in positive actions easier to interpret: more experienced players typically obtain a larger offensive role, mechanically raising successful-action counts without necessarily improving per-action efficiency, supported by the flat trend in shot efficiency and goals. Throwing velocity, CMJ, with-ball speed and agility, Yo-Yo endurance, plank, and curl-ups all improved significantly, alongside two coordination indicators (Section 4.3), a coherent picture of a squad becoming fitter and more skilled as it matured.

The isolated 2026 season did not continue this trajectory uniformly. Compared with the trend prediction, 2026 was markedly worse on endurance and strength (Yo-Yo IE1, flexed-arm hang, plank, curl-ups), but markedly better on coordination, with a smaller running–dribbling gap on all four indicators, and somewhat higher on positive actions and body composition. With only a third of the 2022 roster retained, this most plausibly reflects a genuinely different, more coordinated but less conditioned playing group, cautioning against extrapolating 2010–2022 trends without re-verifying against the current roster.

To test whether years with better fitness also showed better game performance independent of trend, we correlated yearly team-mean fitness and game-quality indicators across 13 continuous seasons (Table 6), then recomputed each significant association on residuals after removing the year trend. Seven of 22 survived, all involving sprint, change-of-direction, or agility speed, more coherent than when 2026 was included, where only one survived. This converges with Section 4.3: sprint and agility speed, not endurance, strength, or coordination, show the most consistent, trend-independent relationship with performance.

Taken together, these findings point to a clear training priority. Sprint, change-of-direction, and agility speed consistently track with game-quality performance, not general endurance, strength, or coordination, and should receive proportionally more attention if the goal is to improve match outcomes. Endurance and strength remain important for physical resilience and injury prevention, but the data give little evidence that further gains here translate into better on-court performance.

The 2026 discontinuity adds a practical caution. A roster can improve in one domain (coordination) while regressing in another (endurance, strength) within a single season, especially with substantial turnover, and neither shift is automatically reflected in game outcomes unless speed and agility are specifically monitored. Coaches should treat each substantially changed roster as its own baseline rather than assuming it continues past trends. Regular within-season testing keeps training aligned with performance drivers [2].

### 4.6. Limitations

Several limitations should be considered. First, the analysis is observational and correlational; no causal claims can be made about fitness driving game performance, since a player’s output could also influence how she is trained or how much she plays. Second, career-average values combine data across different seasons and ages, which may mask within-player change. Third, subgroup sizes for some positions (Pivot, N = 3) and for goalkeepers (N = 7) were small, limiting statistical power and making the goalkeeper-specific correlations in Section 4.3 especially vulnerable to sampling variability; a single data point can noticeably shift a correlation computed on so few observations. Fourth, the year-level partial-correlation analysis in Section 4.4, though improved over the pooled 14-season version, still rests on only 13 yearly points and should be treated as suggestive rather than confirmatory, particularly near the *p* = 0.05 threshold. Fifth, the 3-year gap before 2026 and its near-complete roster turnover mean any trend-based prediction for that season should be treated with caution; we cannot distinguish a true training- or roster-driven shift from a single unusual season. Finally, the game-quality indicators used here are relatively coarse and do not capture shot type, defensive contribution, or contextual factors such as opponent strength, known to influence handball performance indicators [45].

### 4.7. Practical Applications

The normative values established here, as 95% confidence intervals and percentiles, give coaches practical benchmarks for evaluating players’ physical preparedness and game performance, supporting the identification of individual strengths and weaknesses and the design of evidence-based training. Because preparedness and performance change continuously through the season, coaches should regularly monitor both and adjust training accordingly.

The fitness indicators identified here as most strongly related to game performance may help coaches prioritize training objectives and allocate training time more effectively. The team’s playing model should be adapted to players’ current preparedness and capabilities rather than requiring conformity to a fixed tactical system, particularly important in semi-professional women’s handball, where position-specific abilities and squad composition often constrain tactical choices.

## 5. Conclusions

This study characterized the long-term fitness and game-quality profile of a semi-professional female handball team across 14 competitive seasons. Physical fitness qualities were broadly typical for the sport but were sub-elite relative to published elite comparisons, whereas competitive-activity indicators were level-relative rather than absolute markers of quality. Competitive experience and handball-specific abilities, namely throwing velocity and sprint/agility speed, were the strongest and most consistent determinants of game-quality performance, while general endurance, strength, and coordination were not. Universal players emerged as a practically important solution to the positional shortages characteristic of semi-professional squads. Fitness and game-quality indicators changed meaningfully across the continuous 2010–2022 period, but the isolated 2026 season did not continue this trajectory, underscoring that trend-based extrapolation should not replace direct testing of the current roster. These findings provide normative reference values and identify a small set of trainable qualities that coaches can prioritize to translate physical preparation into match performance in semi-professional women’s handball.

## Figures and Tables

**Table 1:** Descriptive statistics (N = 45 players, career averages). Mean, SD, 95% CI, percentiles (P10–P90). Shot efficiency (n = 38 outfield); goalkeeper equivalent in Table 2.

Indicator	N	Mean	SD	95% CI	P10	P25	Median (P50)	P75	P90
**I. Experience**									
Age, years	45	23.44	4.63	22.05–24.83	19.5	20.5	22	25.57	28.06
Experience in championship, years	45	5.45	3.61	4.36–6.53	2.2	3	4.5	6.67	8.83
**II. Game performance**									
Goals per match	38	3.4	1.81	2.80–4.00	1.55	1.91	3.18	4.51	5.36
Shot efficiency, %	38	55.74	6	53.76–57.71	48.15	50.97	56.65	59.69	62.17
Positive actions, points	38	9.72	2.73	8.82–10.61	6.57	7.81	9.94	11.28	12.4
Negative actions, points	38	9.32	1.76	8.74–9.90	7.09	7.75	9.5	10.17	11.6
Net balance (pos.-neg.), points	38	0.4	1.85	−0.21–1.01	−1.81	−0.66	0.3	1.46	2.49
**III. Specific fitness**									
Ball throw velocity (3-step), km/h	44	80.98	6.41	79.03–82.93	74	77	81	85.15	89.64
Ball throw velocity (jump), km/h	43	79.94	6.83	77.84–82.04	72.2	75.17	79.4	85	88.45
Ball throw velocity (7 m), km/h	43	76.77	5.81	74.99–78.56	69.88	74	77	80	85
**IV. Power**									
Countermovement jump (CMJ), cm	44	43.46	4.84	41.99–44.93	37.9	40.07	42.43	46.72	49.57
**V. Speed**									
Speed 5 m, m/s	44	4.44	0.37	4.32–4.55	3.98	4.12	4.36	4.8	4.95
Speed 5 m with ball, m/s	45	4.19	0.36	4.08–4.30	3.74	3.92	4.2	4.4	4.69
Speed 20 m, m/s	45	5.82	0.27	5.74–5.90	5.52	5.65	5.82	6.04	6.1
Speed 20 m with ball, m/s	45	5.55	0.26	5.48–5.63	5.28	5.43	5.57	5.73	5.84
Flying speed 15 m, m/s	44	6.49	0.28	6.41–6.57	6.18	6.38	6.5	6.68	6.79
Flying speed 15 m with ball, m/s	45	6.24	0.3	6.15–6.34	5.97	6.11	6.22	6.43	6.5
**VI. Agility**									
Agility test, s	40	14.79	1.05	14.45–15.12	13.24	14.2	14.84	15.32	16.1
Agility test with ball, s	40	16.66	1.13	16.29–17.02	15.13	16.01	16.65	17.32	18.07
**VII. Coordination/Dribble Deficit**									
Coordination 5 m, %	44	6.69	3.64	5.59–7.80	1.35	4.12	7.06	8.79	11.47
Coordination 20 m, %	45	4.91	2.91	4.04–5.79	3.21	3.63	4.57	5.47	7.88
Coordination flying 15 m, %	44	3.9	4.54	2.52–5.28	−0.04	2.17	3.74	5.49	8.46
Coordination agility, %	40	12.04	5.86	10.17–13.92	9.81	10.55	11.63	13.57	16.27
**VIII. Strength**									
Flexed-arm hang, s	41	37.41	16.53	32.20–42.63	21.1	26.07	30.1	43.55	65.89
Plank, s	42	148.04	113.39	112.71–183.38	58	65.9	105.9	196.77	290.28
Sit-ups in 30 s, reps	42	31.86	3.44	30.78–32.93	28.41	29	31.3	34.35	36.35
Curl-up in 60 s, reps	43	93.38	7.54	91.06–95.70	86.2	89	91.2	98.29	101.67
**IX. Endurance**									
Yo-Yo IE1, m	42	2401.47	811.97	2148.44–2654.50	1334	1870	2360	2639	3384
3 km run, s	37	895.46	69.68	872.22–918.69	808.5	850.8	893.6	947	971.3
VO_2_max, mL/kg/min	43	48.01	3.61	46.90–49.13	44.23	45.74	46.9	48.99	53.86
**X. Body composition**									
Height, cm	45	173.9	5.53	172.24–175.56	168.85	170	173	178	180
Body mass, kg	45	68.28	8.08	65.85–70.71	57.6	63	66.33	73.37	79.16
Body fat, kg	44	15.06	5.15	13.49–16.62	9.46	10.38	15.04	18.27	20.9
BMI, kg/m^2^	45	22.55	2.14	21.90–23.19	20.02	21.08	22.57	23.82	24.94
Body fat, %	44	21.77	5.71	20.04–23.51	15.27	16.88	20.94	26.1	29.07
Fat-free mass, kg	44	53.02	5.26	51.42–54.62	47.69	50.57	53.16	55.78	59.59
Fat-free mass, %	44	78.23	5.71	76.49–79.96	70.93	73.9	79.06	83.12	84.73
Fat mass index, kg/m^2^	44	4.97	1.65	4.47–5.47	3.24	3.6	4.67	6.24	6.87
Fat-free mass index, kg/m^2^	44	17.53	1.36	17.12–17.94	15.99	16.74	17.59	18.73	19.14

**Table 2:** Goalkeeper indicators (described separately from outfield players). Game-action indicators are not recorded for goalkeepers and are omitted here. “Save efficiency, %” is the goalkeeper equivalent of outfield “Shot efficiency, %”, reflecting the share of opponent shots saved.

Indicator	N	Mean	SD	95% CI
**I. Experience**				
Age, years	7	26.21	8.03	20.27–32.16
Experience in championship, years	7	7.93	7.25	2.55–13.30
**II. Game performance**				
Save efficiency, %	7	36.89	5.56	31.75–42.03
**III. Specific fitness**				
Ball throw velocity (3-step), km/h	6	82.75	6.06	76.39–89.11
Ball throw velocity (jump), km/h	5	80.28	5.61	73.31–87.25
Ball throw velocity (7 m), km/h	5	78.84	4.41	73.37–84.31
**IV. Power**				
Countermovement jump (CMJ), cm	7	42.22	3.81	38.70–45.74
**V. Speed**				
Speed 5 m, m/s	7	4.08	0.16	3.93–4.23
Speed 5 m with ball, m/s	7	3.89	0.19	3.71–4.07
Speed 20 m, m/s	7	5.56	0.31	5.27–5.85
Speed 20 m with ball, m/s	7	5.41	0.31	5.12–5.70
Flying speed 15 m, m/s	7	6.34	0.49	5.88–6.80
Flying speed 15 m with ball, m/s	7	6.24	0.47	5.81–6.67
**VI. Agility**				
Agility test, s	4	15.82	0.32	15.32–16.33
Agility test with ball, s	4	18	0.48	17.24–18.77
**VII. Coordination/Dribble Deficit**				
Coordination 5 m, %	7	5.86	3.41	2.70–9.02
Coordination 20 m, %	7	2.83	2.91	0.13–5.52
Coordination flying 15 m, %	7	0.67	4.64	−3.62–4.96
Coordination agility, %	4	13.85	4.43	6.80–20.91
**VIII. Strength**				
Flexed-arm hang, s	5	37.8	18.6	14.71–60.89
Plank, s	6	205.92	121.25	78.68–333.17
Sit-ups in 30 s, reps	6	32.94	5.25	27.43–38.45
Curl-up in 60 s, reps	6	88.45	6.65	81.47–95.43
**IX. Endurance**				
Yo-Yo IE1, m	4	2319	807.24	1034.51–3603.49
3 km run, s	5	927.83	61.92	850.95–1004.71
VO_2_max, mL/kg/min	5	45.39	1.3	43.77–47.00
**X. Body composition**				
Height, cm	7	178	4.08	174.22–181.78
Body mass, kg	7	77.24	5.68	71.99–82.50
Body fat, kg	6	20.32	6.31	13.70–26.94
BMI, kg/m^2^	7	24.42	2.25	22.34–26.50
Body fat, %	6	26.08	6.6	19.15–33.00
Fat-free mass, kg	6	56.86	4.22	52.43–61.29
Fat-free mass, %	6	73.92	6.6	67.00–80.85
Fat mass index, kg/m^2^	6	6.44	2.1	4.24–8.65
Fat-free mass index, kg/m^2^	6	17.97	1.47	16.43–19.50

**Table 3:** Indicator means (95% CI) by playing position and significance of differences (ANOVA). *Back, Wing, Pivot, Universal; goalkeepers excluded. Each cell: mean (95% CI). F and p are one-way ANOVA results. Last column shows which position pairs differ significantly (Tukey HSD).*

Indicator	Back	Wing	Pivot	Universal	F	*p*	Significant Pairwise Differences (Tukey)
**I. Experience**							
Age, years	25.53 (23.24–27.81)	20.78 (19.65–21.91)	20.61 (17.29–23.93)	22.99 (20.06–25.92)	6.26	0.002	Back vs. Wing
Experience in championship, years	6.76 (5.50–8.01)	3.86 (2.68–5.04)	2.83 (0.25–5.42)	4.64 (3.26–6.02)	6.6	0.001	Back vs. Wing; Back vs. Pivot
**II. Game performance**							
Goals per match	4.50 (3.33–5.68)	2.46 (1.80–3.13)	1.71 (1.19–2.24)	3.79 (2.49–5.09)	5.32	0.004	Back vs. Wing; Back vs. Pivot
Shot efficiency, %	58.61 (55.40–61.82)	52.76 (49.61–55.90)	54.81 (43.20–66.42)	56.34 (50.29–62.39)	2.55	0.072	
Positive actions, points	11.35 (9.83–12.86)	7.88 (6.84–8.92)	6.82 (2.90–10.74)	11.36 (10.15–12.58)	9.94	0	Back vs. Wing; Back vs. Pivot; Wing vs. Universal; Pivot vs. Universal
Negative actions, points	10.05 (9.21–10.89)	8.15 (7.43–8.87)	8.08 (4.11–12.05)	10.71 (9.12–12.30)	7.19	0.001	Back vs. Wing; Wing vs. Universal
Net balance (pos.-neg.), points	1.30 (0.25–2.35)	−0.27 (−1.21–0.67)	−1.26 (−4.94–2.42)	0.65 (−0.99–2.29)	2.95	0.046	
**III. Specific fitness**							
Ball throw velocity (3-step), km/h	83.25 (79.53–86.97)	78.10 (75.41–80.78)	77.53 (63.34–91.73)	82.14 (74.16–90.13)	1.96	0.138	
Ball throw velocity (jump), km/h	83.10 (79.32–86.87)	76.99 (73.95–80.03)	74.16 (62.44–85.88)	81.76 (73.42–90.10)	3	0.044	
Ball throw velocity (7 m), km/h	79.99 (76.75–83.24)	74.09 (71.86–76.31)	72.77 (58.48–87.05)	75.95 (68.89–83.00)	3.3	0.032	Back vs. Wing
**IV. Power**							
Countermovement jump (CMJ), cm	45.96 (42.62–49.31)	43.74 (41.13–46.35)	37.44 (32.94–41.94)	42.07 (38.99–45.14)	3.16	0.038	Back vs. Pivot
**V. Speed**							
Speed 5 m, m/s	4.58 (4.40–4.76)	4.57 (4.34–4.80)	4.12 (4.07–4.18)	4.39 (4.00–4.77)	1.79	0.168	
Speed 5 m with ball, m/s	4.28 (4.12–4.44)	4.34 (4.11–4.56)	3.79 (3.45–4.12)	4.20 (3.84–4.57)	2.24	0.102	
Speed 20 m, m/s	5.91 (5.80–6.03)	5.93 (5.79–6.08)	5.52 (5.28–5.77)	5.78 (5.65–5.92)	3.82	0.018	Back vs. Pivot; Wing vs. Pivot
Speed 20 m with ball, m/s	5.65 (5.54–5.77)	5.61 (5.47–5.75)	5.37 (5.18–5.56)	5.46 (5.17–5.75)	1.95	0.14	
Flying speed 15 m, m/s	6.51 (6.41–6.62)	6.60 (6.47–6.73)	6.23 (5.84–6.62)	6.49 (6.31–6.67)	3.04	0.043	Wing vs. Pivot
Flying speed 15 m with ball, m/s	6.35 (6.18–6.52)	6.23 (6.11–6.34)	6.24 (5.84–6.64)	6.08 (5.76–6.39)	1.66	0.194	
**VI. Agility**							
Agility test, s	14.58 (14.04–15.13)	14.64 (14.01–15.27)	15.69 (13.02–18.36)	14.46 (13.31–15.62)	1.1	0.364	
Agility test with ball, s	16.35 (15.71–17.00)	16.44 (15.87–17.02)	17.59 (14.66–20.51)	16.44 (15.25–17.62)	1.12	0.356	
**VII. Coordination/Dribble Deficit**							
Coordination 5 m, %	7.69 (5.57–9.82)	6.27 (3.87–8.68)	8.94 (−1.51–19.39)	5.53 (3.01–8.04)	0.95	0.426	
Coordination 20 m, %	4.57 (3.29–5.86)	6.05 (4.77–7.33)	2.89 (−3.23–9.02)	6.27 (2.40–10.14)	1.81	0.164	
Coordination flying 15 m, %	3.33 (1.02–5.65)	5.75 (3.96–7.54)	−0.16 (−5.98–5.66)	6.25 (0.51–12.00)	2.56	0.072	
Coordination agility, %	12.45 (11.70–13.19)	12.51 (8.68–16.34)	12.10 (7.77–16.43)	9.36 (−0.68–19.41)	0.47	0.704	
**VIII. Strength**							
Flexed-arm hang, s	44.62 (31.88–57.35)	35.57 (27.76–43.38)	25.71 (18.54–32.89)	33.50 (18.64–48.36)	1.52	0.229	
Plank, s	207.68 (108.93–306.42)	99.17 (68.15–130.19)	66.86 (34.93–98.79)	128.74 (61.75–195.72)	3.03	0.044	
Sit-ups in 30 s, reps	33.34 (31.27–35.42)	30.87 (29.44–32.31)	28.48 (27.37–29.60)	31.79 (28.64–34.94)	2.92	0.049	
Curl-up in 60 s, reps	95.53 (91.10–99.96)	92.69 (88.22–97.17)	88.97 (84.52–93.41)	96.89 (89.54–104.25)	1.14	0.347	
**IX. Endurance**							
Yo-Yo IE1, m	2617.94 (2061.72–3174.15)	2305.16 (1904.33–2705.98)	1662.22 (762.84–2561.61)	2525.09 (1777.03–3273.16)	1.27	0.299	
3 km run, s	886.40 (838.03–934.78)	883.62 (843.75–923.48)	919.80 (833.40–1006.20)	899.90 (811.31–988.50)	0.19	0.901	
VO_2_max, mL/kg/min	49.52 (47.40–51.64)	48.02 (46.06–49.99)	46.63 (43.53–49.73)	47.44 (43.02–51.87)	0.86	0.471	
**X. Body composition**							
Height, cm	174.43 (170.75–178.11)	170.18 (168.07–172.30)	175.00 (163.62–186.38)	175.71 (170.98–180.45)	2.54	0.073	
Body mass, kg	68.04 (63.33–72.76)	63.06 (59.77–66.35)	72.28 (54.15–90.42)	68.51 (62.09–74.94)	2.2	0.106	
Body fat, kg	14.91 (12.26–17.55)	12.10 (9.97–14.23)	19.48 (10.77–28.20)	14.87 (10.75–18.98)	2.98	0.045	Wing vs. Pivot
BMI, kg/m^2^	22.31 (21.23–23.39)	21.81 (20.57–23.04)	23.61 (17.96–29.26)	22.17 (20.57–23.77)	0.71	0.555	
Body fat, %	21.63 (18.65–24.60)	19.16 (15.95–22.36)	26.84 (20.99–32.68)	21.44 (16.93–25.95)	1.97	0.137	
Fat-free mass, kg	53.14 (49.83–56.44)	51.00 (47.86–54.14)	52.80 (42.41–63.19)	53.65 (49.76–57.54)	0.55	0.65	
Fat-free mass, %	78.37 (75.40–81.35)	80.84 (77.64–84.05)	73.16 (67.32–79.01)	78.56 (74.05–83.07)	1.97	0.137	
Fat mass index, kg/m^2^	4.89 (4.02–5.77)	4.21 (3.40–5.02)	6.35 (4.01–8.68)	4.80 (3.52–6.08)	1.99	0.133	
Fat-free mass index, kg/m^2^	17.42 (16.84–17.99)	17.60 (16.57–18.63)	17.26 (13.23–21.30)	17.37 (16.38–18.36)	0.08	0.971	

**Table 4:** Correlations between age/experience, fitness indicators, and game-quality indicators (Pearson r). Each cell shows the Pearson correlation coefficient (*r*). * *p* < 0.05; ** *p* < 0.01. *N*, 95% CI, and exact *p* for every pair are given in Supplementary Table S1.

Indicator	Goals per Match	Shot Efficiency, %	Positive Actions, Points	Negative Actions, Points	Net Balance (Pos.-Neg.), Points
**I. Experience**					
Age, years	0.34 *	−0.02	0.34 *	0.19	0.32 *
Experience in championship, years	0.51 **	0.02	0.44 **	0.20	0.46 **
**III. Specific fitness**					
Ball throw velocity (3-step), km/h	0.38 *	0.06	0.48 **	0.33 *	0.39 *
Ball throw velocity (jump), km/h	0.44 **	0.11	0.50 **	0.38 *	0.37 *
Ball throw velocity (7 m), km/h	0.36 *	0.07	0.44 **	0.28	0.39 *
**IV. Power**					
Countermovement jump (CMJ), cm	0.13	0.05	0.29	0.04	0.39 *
**V. Speed**					
Speed 5 m, m/s	0.07	0.26	0.05	−0.16	0.25
Speed 5 m with ball, m/s	0.24	0.26	0.26	0.08	0.30
Speed 20 m, m/s	0.28	0.28	0.26	−0.02	0.41 *
Speed 20 m with ball, m/s	0.46 **	0.19	0.32 *	0.16	0.32 *
Flying speed 15 m, m/s	0.16	0.05	0.14	0.00	0.19
Flying speed 15 m with ball, m/s	0.46 **	0.03	0.22	0.15	0.18
**VI. Agility**					
Agility test, s	−0.41 *	−0.18	−0.13	−0.21	0.03
Agility test with ball, s	−0.41 *	−0.17	−0.21	−0.19	−0.09
**VII. Coordination/Dribble Deficit**					
Coordination 5 m, %	−0.18	−0.09	−0.28	−0.19	−0.19
Coordination 20 m, %	−0.31	0.04	−0.09	−0.18	0.03
Coordination flying 15 m, %	−0.23	0.13	0.08	−0.10	0.22
Coordination agility, %	0.00	−0.09	−0.14	−0.11	−0.08
**VIII. Strength**					
Flexed-arm hang, s	0.12	0.21	0.16	−0.06	0.29
Plank, s	0.34 *	0.17	0.27	0.27	0.10
Sit-ups in 30 s, reps	0.40 *	0.13	0.39 *	0.27	0.26
Curl-up in 60 s, reps	−0.12	0.24	−0.02	−0.17	0.12
**IX. Endurance**					
Yo-Yo IE1, m	0.10	−0.00	0.27	0.08	0.32 *
3 km run, s	0.00	−0.04	0.00	0.20	−0.21
VO_2_max, mL/kg/min	0.34 *	0.16	0.20	0.15	0.15
**X. Body composition**					
Height, cm	0.17	−0.06	0.20	0.24	0.06
Body mass, kg	0.15	−0.17	0.26	0.41 **	−0.01
Body fat, kg	0.09	−0.04	0.24	0.35 *	0.02
BMI, kg/m^2^	0.06	−0.16	0.19	0.34 *	−0.05
Body fat, %	0.04	0.03	0.19	0.24	0.04
Fat-free mass, kg	0.13	−0.20	0.16	0.28	−0.04
Fat-free mass, %	−0.04	−0.03	−0.19	−0.24	−0.04
Fat mass index, kg/m^2^	0.06	−0.02	0.21	0.30	0.02
Fat-free mass index, kg/m^2^	0.03	−0.21	0.05	0.17	−0.09

Full statistical details for every fitness–game-quality pair (N, 95% CI, and exact *p*) are provided in Supplementary Table S1.

**Table 5:** Trend over 13 continuous seasons (2010–2022); 2026 excluded due to the gap and roster turnover. *r* (vs year), Pearson correlation with calendar year; *F* (ANOVA), one-way ANOVA across seasons; Predicted 2026, extrapolation of the 2010–2022 trend; Actual − Predicted, difference between the observed and predicted 2026 means. * *p* < 0.05; ** *p* < 0.01; *** *p* < 0.001.

Indicator	N (2010–2022)	r (vs. Year)	Sig.	*p* (Trend)	F (ANOVA)	*p* (ANOVA)	Sig.	N (2026)	Mean (2026, Actual)	Predicted 2026 (From 2010–2022 Trend)	Actual–Predicted
**I. Experience**											
Age, years	162	0.27	***	0.0	1.4	0.173		9	24.94	27.52	−2.58
Experience in championship, years	162	0.38	***	0.0	2.39	0.007	**	9	6.89	10.38	−3.49
**II. Game performance**											
Goals per match	144	0.05		0.564	0.89	0.558		7	4.66	3.88	0.78
Shot efficiency, %	162	0.04		0.655	1.26	0.25		9	51.83	56.11	−4.28
Positive actions, points	144	0.36	***	0.0	2.29	0.011	*	7	13.54	12.81	0.73
Negative actions, points	144	0.16		0.062	3.22	0.0	***	7	12.39	10.06	2.33
**III. Specific fitness**											
Ball throw velocity (3-step), km/h	157	0.13		0.093	2.62	0.003	**	9	80.78	84.0	−3.22
Ball throw velocity (jump), km/h	153	0.27	***	0.001	3.14	0.001	***	9	80.0	86.48	−6.48
Ball throw velocity (7 m), km/h	153	0.2	*	0.015	2.73	0.002	**	9	76.33	80.8	−4.47
**IV. Power**											
CMJ, cm	159	0.18	*	0.021	1.68	0.077		9	46.21	45.25	0.96
**V. Speed**											
Speed 5 m, m/s	162	−0.09		0.274	0.46	0.934		7	4.16	4.4	−0.24
Speed 5 m with ball, m/s	162	−0.06		0.456	0.54	0.888		9	4.23	4.14	0.09
Speed 20 m, m/s	162	−0.03		0.671	0.28	0.992		9	5.87	5.78	0.09
Speed 20 m with ball, m/s	162	−0.17	*	0.034	0.8	0.651		9	5.66	5.42	0.24
Flying speed 15 m, m/s	162	0.02		0.784	0.32	0.984		7	6.73	6.47	0.26
Flying speed 15 m with ball, m/s	162	−0.21	**	0.009	2.11	0.019	*	9	6.39	6.04	0.35
**VI. Agility**											
Agility test, s	158	0.15		0.053	0.71	0.742		5	14.26	15.23	−0.97
Agility test with ball, s	157	0.31	***	0.0	1.5	0.131		5	16.6	17.67	−1.07
**VII. Coordination/Dribble Deficit**											
Coordination 5 m, %	162	−0.05		0.546	0.52	0.9		7	1.21	6.39	−5.18
Coordination 20 m, %	162	0.23	**	0.003	1.47	0.142		9	3.41	6.9	−3.49
Coordination flying 15 m, %	162	0.24	**	0.002	1.88	0.041	*	7	2.23	7.19	−4.96
Coordination agility, %	158	0.07		0.354	0.94	0.508		5	14.26	13.95	0.31
**VIII. Strength**											
Flexed-arm hang, s	150	0.16		0.051	1.13	0.344		8	33.34	50.84	−17.5
Plank, s	156	0.19	*	0.017	1.01	0.447		8	160.0	229.7	−69.7
Sit-ups in 30 s, reps	156	0.14		0.072	0.45	0.94		8	33.88	33.92	−0.04
Curl-up in 60 s, reps	156	0.27	***	0.001	2.13	0.018	*	9	85.56	100.42	−14.86
**IX. Endurance**											
Yo-Yo IE1, m	153	0.39	***	0.0	2.89	0.001	**	8	2265.0	3454.42	−1189.42
3 km run, s	148	0.15		0.073	1.34	0.204		6	959.0	911.55	47.45
VO_2_max, mL/kg/min	153	−0.03		0.758	0.48	0.924		9	48.04	48.24	−0.2
**X. Body composition**											
Height, cm	162	−0.11		0.168	0.71	0.736		9	172.33	172.55	−0.22
Body mass, kg	162	0.11		0.183	0.99	0.461		9	73.62	70.14	3.48
Body fat, kg	156	0.11		0.17	0.56	0.87		9	20.46	16.15	4.31

**Table 6:** Yearly team-mean correlations across 13 continuous seasons (2010–2022). Partial *r* and partial *p* were obtained after removing each series’ linear trend against year. Sig., significance of raw *r*; Partial Sig., significance of partial *r*. * *p* < 0.05; ** *p* < 0.01; *** *p* < 0.001. The full 112-pair set is given in Supplementary Table S2.

Fitness Indicator	Game-Quality Indicator	N (Years)	r	*p*	Sig.	Partial r (Year-adj.)	Partial *p*	Partial Sig.
Agility test with ball, s	Positive actions, points	13	0.92	0.000	***	0.58	0.036	*
Speed 20 m with ball, m/s	Positive actions, points	13	−0.85	0.000	***	−0.67	0.013	*
Experience in championship, years	Positive actions, points	13	0.77	0.002	**	−0.33	0.270	
Speed 20 m with ball, m/s	Negative actions, points	13	−0.78	0.002	**	−0.82	0.001	***
Agility test, s	Positive actions, points	13	0.75	0.003	**	0.50	0.084	
Yo-Yo IE1, m	Positive actions, points	13	0.75	0.003	**	−0.05	0.871	
Sit-ups in 30 s, reps	Positive actions, points	13	0.75	0.003	**	0.23	0.448	
Age, years	Positive actions, points	13	0.74	0.004	**	−0.16	0.606	
Coordination 20 m, %	Positive actions, points	13	0.74	0.004	**	0.30	0.312	
Flying speed 15 m, m/s	Goals per match	13	−0.70	0.008	**	−0.74	0.004	**
Flying speed 15 m with ball, m/s	Negative actions, points	13	−0.68	0.011	*	−0.63	0.022	*
Flying speed 15 m with ball, m/s	Positive actions, points	13	−0.68	0.011	*	−0.43	0.146	
Coordination flying 15 m, %	Positive actions, points	13	0.67	0.012	*	0.22	0.466	
Flying speed 15 m, m/s	Negative actions, points	13	−0.66	0.015	*	−0.73	0.005	**
Speed 20 m, m/s	Negative actions, points	13	−0.61	0.025	*	−0.57	0.043	*
Height, cm	Negative actions, points	13	−0.61	0.027	*	−0.55	0.053	
3 km run, s	Negative actions, points	13	0.61	0.028	*	0.55	0.051	
Body fat, kg	Positive actions, points	13	0.60	0.029	*	0.34	0.260	
Coordination 20 m, %	Negative actions, points	13	0.57	0.044	*	0.49	0.086	
Speed 5 m, m/s	Positive actions, points	13	−0.56	0.046	*	−0.30	0.312	
Flexed-arm hang, s	Positive actions, points	13	0.56	0.048	*	0.14	0.656	
Curl-up in 60 s, reps	Positive actions, points	13	0.55	0.050	*	−0.23	0.458	

## Data Availability

The data supporting the findings of this study are publicly available.

## Supplementary Materials

The following supporting information can be downloaded at: https://www.mdpi.com/article/10.3390/sports14100469/s1, Table S1: Full detail for every fitness/age/experience–game-quality pair; Table S2: Full set of 112 year-level (team-average) correlations between fitness/age/experience indicators and game-quality indicators across the 13 continuously observed seasons (2010–2022; 2026 excluded, see Section 3.4).
